# Real time monitoring and evaluation of the inhibition effect of fucoxanthin against α-amylase activity by using QCM-A

**DOI:** 10.3389/fnut.2022.1110615

**Published:** 2023-01-12

**Authors:** Shipeng Yin, Evi Amelia Siahaan, Liqiong Niu, Mario Shibata, Yuanfa Liu, Tomoaki Hagiwara

**Affiliations:** ^1^State Key Laboratory of Food Science and Technology, National Engineering Laboratory for Cereal Fermentation Technology, School of Food Science and Technology, Collaborative Innovation Center of Food Safety and Quality Control in Jiangsu Province, National Engineering Research Center for Functional Food, Jiangnan University, Wuxi, China; ^2^Department of Food Science and Technology, Tokyo University of Marine Science and Technology, Tokyo, Japan; ^3^Research Centre for Marine and Land Bioindustry, National Research and Innovation Agency, Bogor, Indonesia; ^4^School of Life Sciences, Guangzhou University, Guangzhou, China

**Keywords:** QCM-A, amylose-immobilized, enzyme kinetic parameters, fucoxanthin, α-amylase inhibitor

## Abstract

The main symptoms of diabetes are hyperglycemia and insulin resistance. The inhibition of the starch digestion enzymes could effectively regulate starch digestion and glucose absorption, thereby slowing or treating the symptoms of postprandial hyperglycemia. Herein, we used fucoxanthin isolated from *Undaria pinnatifida* stems, as α-amylase inhibitor, and monitored the interactions of both biomolecules by using quartz crystal microbalance-admittance (QCM-A) instrument. All the processes of α-amylase hydrolysis of starch were also dynamically tracked by using amylose-immobilized QCM technology. In our work, we found that the kinetic parameter (*k*_off_, *k*_on_, and *k*_cat_) values obtained by the QCM-A analysis were relatively consistent compared to the kinetic parameter values obtained by the conventional Michaelis–Menten analysis. For the inhibitory reactions, the results showed that fucoxanthin significantly reduced the activity of α-amylase in a dose-dependent manner. The QCM-A technology shown to be an excellent approach in obtaining comprehensive and accurate kinetic parameters, thereby providing real and accurate data for kinetic studies. It is helpful to clarify the mechanism of action of fucoxanthin on α-amylase, which further proved the potential of fucoxanthin to improve and treat postprandial hyperglycemia.

## Highlights

- A method was established in QCM-A to monitor and quantify the inhibition of α-amylase hydrolysis of amylose by fucoxanthin in real-time.
- Comprehensive and precise enzyme kinetic parameters can be obtained by QCM technology.
- Fucoxanthin significantly reduced the amylose hydrolyzing activity of α-amylase in a dose-dependent manner.
- The inhibition type of fucoxanthin on α-amylase is mixed inhibition.

## 1. Introduction

Diabetes mellitus (DM), as a global chronic disease, has increased rapidly in recent years and tends to attack young generation (1, 2). Hyperglycemia and insulin resistance are the manifestations of DM, that impair human vital organs including the cardiovascular organ, kidneys, eyes, and nerves. It has been reported that DM can lead to numerous severe complications, therefore it has become the global burden disease in the 21st century and has received serious attention. Controlling the glucose level in blood remains the most effective approach for diabetic prevention (3). The glucose in blood originates from the hydrolysis of carbohydrates and it is catalyzed by digestive enzymes, such as α-amylase and α-glucosidase (4). These two key enzymes play important roles in converting polysaccharides and oligosaccharide into simple sugars and lead to the rapid absorption of glucose in human body (3, 5). Therefore, the inhibition of α-amylase and α-glucosidase activities is considered as an effective method to retard the carbohydrate digestibility, thus reducing the rate of glucose absorption into the blood. Numerous synthetic inhibitors such as acarbose, miglitol, and voglibose, are often clinically to suppress the activity of carbohydrate digestive enzymes. However, the use of synthetic inhibitors causes adverse gastrointestinal effects such as diarrhea, flatulence, and abdominal pain, and liver function disorder (6–8). Thus, the use of enzyme inhibitors derived from natural resources seems to be the best alternative to the synthetic.

Marine micro- and macroalgae are known as natural sources of useful bioactive compounds including phenolics, polysaccharides, and carotenoids. In particular algae-derived carotenoids, fucoxanthin, have gained more attention for their ability to improve the symptoms of postprandial hyperglycemia, by inhibiting the actions of α-amylase and α-glucosidase (9–14). Numerous previous works have reported that the interaction between fucoxanthin and α-amylase shown great potential for reducing postprandial blood glucose, yet the mechanisms of interaction between these two molecules is not clearly understood (11, 15–22). Understanding the fucoxanthin-α-amylase interactions is important and will be of value for the development of new diabetes drugs as well as antidiabetic supplements. Here, we demonstrated quartz crystal microbalance (QCM) based on admittance analysis (QCM-A), a sensitive mass sensor, novel method, and simple concept, for understanding the kinetics of enzymatic hydrolysis of glucose polymer with an α-amylase and observing how the seaweed fucoxanthin affected on the actions of α-amylase.

Quartz crystal microbalance is a mass sensing technology characterized by ultra-sensitivity and high resolution, which is approximately 100 times more sensitive than typical precision analytical balances, so it can be used to determine mass changes at a nanogram level or less than 1 μg cm^–2^ mass density (23–27). The typical QCM is made of a thin AT-cut quartz crystal and placed between two electrodes linked to an oscillator, which, when energized, produces a piezoelectric effect that has very stable oscillations at a resonant frequency. Therefore, the most significant feature of QCM is that it can perform real-time measurements of changes in quality and viscosity (28). QCM-A is a new generation of QCM, that can evaluate the physical properties, including hydrodynamic water (bound and vibrated water) mass and the viscoelasticity, of loading materials/biomolecules on the QCM plate in aqueous solutions (29). In QCM-A measurements, the viscosity contribution can be estimated separately by measuring the resonance frequency and the energy dissipation (D factor) that indicates energy loss from the viscous components of solution and the loading materials (30). Recently, QCM-A has been widely employed to identify and quantify various interactions between biomolecules such as DNA transcription, assembly of protein complexes, protein-polysaccharide interactions, thermodynamic and kinetic properties of biomolecular interactions on cell surface, and enzyme-substrate interactions (29, 31–33).

The Michaelis–Menten equation is a common model used for understanding the kinetic expression of single substrate enzyme mechanisms, as shown in Equation 1. This kinetic model presumed a steady-state condition, in which the concentration of the enzyme-substrate (ES) is assumed to be constant during the reaction, because of the relative difficulty of determining the concentration of the ES complex (31, 34). The reaction rate was simply measured as the initial rate (*v*_0_) of the product increase and product release in defining *K*_m_ could be possible to obtain according to a minimal model of Michaelis–Menten (Equation 2). However, *K*_m_ is a complex value containing *k*_cat_, *k*_on_, and *k*_off_ (Equation 3), and resembles the dissociation constant (*K*_d_) only when *k*_off_ ≫ *k*_cat_, and *K*_d_ is a parameter not measured in real time solution reactions, represents the rates of enzyme binding and release thus allow a better understanding of ES interactions (31).


(1) $$\text{E}+\text{S}\,\begin{array}{c} {\text{k}}_{\text{on}}\\ ⇌\\ {\text{k}}_{\text{off}} \end{array}\,\text{ES}\overset{k_{\text{cat}}}{⟶}\text{E}+\text{P}$$

(2) $$v_{0}=\frac{k_{\text{cat}}\,{\left[\text{E}\right]}_{0}\,{\left[\text{S}\right]}_{0}}{{\left[\text{S}\right]}_{0}+K_{m}}$$

where,


(3) $$K_{m}=\frac{k_{\text{off}}+k_{\text{cat}}}{k_{\text{on}}}$$

However, to effectively ensure the enzyme mechanism above, especially the kinetic mechanism, the best solution is still to determine the kinetic parameters (enzyme binding and release, *k*_on_ and *k*_off_, and intramolecular hydrolysis rates, *k*_cat_) in real time through the mass changes monitored by using a QCM-A. The results are in good agreement with the classical chemical kinetic theory (35–38). By employing the QCM-A technology, we designed and developed a new scheme and effectively detected the relevant data in the α-amylase catalytic mechanism and its inhibition mechanism as well.

**FIGURE 1:**
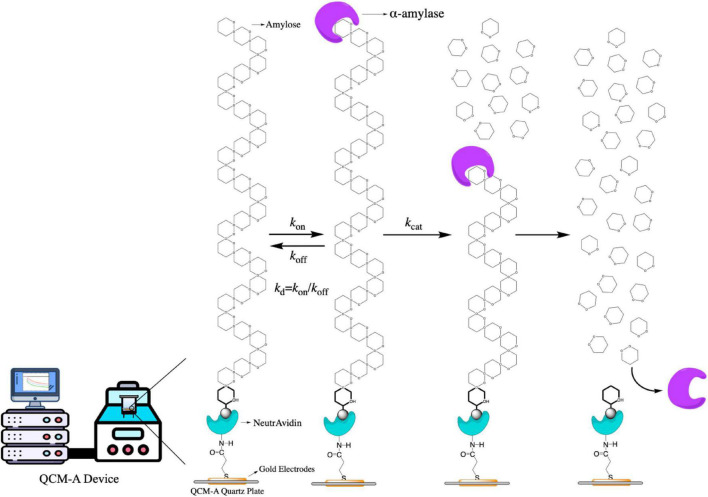
The scheme of the α-amylase mechanism for amylose hydrolysis and the kinetic parameters obtained in this mechanism.

In this study, we described all step of hydrolysis mechanisms (the enzyme binding, release, and hydrolysis). For hydrolyses of amylose as a substrate catalyzed by α-amylase could be obtained quantitatively by using an amylose-immobilized 27 MHz QCM-A due to the formation and decay of the ES complex and the formation of the product could be followed as mass changes on the QCM-A plate as shown in Figure 1. The α-amylase is known to catalyze the release of β-d-glucose (α-1,4 glucan bonds at the branch points of amylose, α-1,4 and α-1,6 glucan bonds at the branch points of amylopectin) from the non-reducing ends of soluble starch (39, 40).

A curve fitting method of single-substrate amylose reactions to obtain the kinetic parameters was also provided in this work. In addition, we could observe all steps of the α-amylase bind to the amylose substrate (mass increase) and hydrolyze to the amylose (mass decrease), because the QCM-A technique could continuously from time dependences of frequency changes detect amounts of both the ES complex and the hydrolysis product with the same physical signal (mass) that can be convertible into a unit on a molar basis. On this basis, we used fucoxanthin (from *Undaria pinnatifida* stems, MW, 658.91) to observe and determine how it affects the activity of α-amylase by kinetic study.

## 2. Materials and methods

### 2.1. Reagents

The α-amylase (from human saliva, EC 3.2.1.1, MW: 60 kDa) and amylose (from corn starch, average MW: ∼16,000) were purchased from Nacalai Tesque Co., Ltd. (Kyoto, Japan). 1-Ethyl-3-[3-(dimethylamino)propyl]carbodiimide (EDC) were purchased from Dojindo, Co., Ltd. (Kumamoto, Japan). *N*-Hydroxysuccinimide (NHS) and ethanolamine were purchased from Tokyo Chemical Industry Co., Ltd. (Tokyo, Japan). 3,3′-Dithiodipropionic Acid and Fucoxanthin standards (PubChem CID: 5281239, 98% purity) were purchased from Fujifilm Wako Pure Chemical Corp. (Osaka, Japan). NeutrAvidin Biotin-Binding Protein was purchased from Thermo Fisher Scientific Inc. (Rockford, IL, USA). All other reagents used in this study were purchased from Fujifilm Wako Pure Chemical Corp. (Osaka, Japan) and used without further purification.

### 2.2. QCM-A setup and calibration in aqueous solutions

The QCM-A instrument (AFFINIX QN pro, Ulvac Inc., Chigasaki, Japan) used in this study has a 500 μL cell with a 27 MHz quartz crystal plate (8.7 mm diameter AT-cut quartz plate with gold electrodes on the bottom of the cell). The effective area is 4.9 mm^2^ and has a stirring function (30). According to previous studies (36, 41), we obtain a Sauerbrey’s (Equation 4), which is suitable for the AT-cut shear mode QCM of air phase as follow:


(4) $$△\,F_{\text{water}}=-\frac{△\,F_{\text{water}}}{△\,F_{\text{air}}}\,\frac{2\,F_{0}^{2}}{A\,\sqrt{\rho_{\text{q}}\,\mu_{\text{q}}}}\,△\,m$$

where Δ*F*_air_: [in Hz] and the measured frequency change in the air phase, *F*_0_: [27 × 106 Hz] and the fundamental frequency of the quartz crystal prior to a mass change, Δ*m*: [in g] and the mass change, *A*: [5.7 mm^2^] and the electrode area, ρ_q_: [2.65 g cm^–3^] and the density of quartz, μ_q_: [2.95 × 10^11^ dyn cm^–2^] and the shear modulus of quartz. In the air phase, the QCM-A was calibrated to change frequency responding to the mass increase of 0.62 ± 0.02 ng cm^–2^ on the electrode.

In the air phase, the mass is expected to increase by 0.62 ng cm^–2^ for every 1 Hz decrease in frequency. However, in the present study, QCM-A was used to investigate the determination of the binding of biomolecules in aqueous solutions, which must consider the effects of bio-molecular hydration (42). Furthermore, according to previous studies, although there is a certain noise level (±1 Hz for 1 h) and bias (±2 Hz) for the 27 MHz QCM at room temperature and buffer solution (36), its sensitivity (0.25–0.30 ng cm^–2^ per −1 Hz) was sufficient to support detection of enzyme binding.

### 2.3. Preparation of amylose-immobilized QCM plates

Before performing the experiments, the Au electrode surface needs to be cleaned with piranha solution (a 3:1 mixture of sulfonic acid and hydrogen peroxide) according to the manufacturer’s operating manual, followed by rinsing with Milli-Q water and air drying (30, 36). Following previous studies, biotinylated amylose with the reducing end reacted with biotinamidocaproyl hydrazide and was immobilized on Neutravidin-immobilized QCM-A with slight modifications (Figure 1) (43, 44). The immobilized amount of biotinylated amylose was kept at 40 ± 5 ng cm^–2^, equivalent to covering 20% of the electrode surface. Although the area covered is small, these spaces are sufficient for monitoring the binding of larger enzyme molecules (45).

In brief, first, purification of amylose to glycan sample by the GlycoClean S cartridges GKI-4276 (Agilent Technologies, Inc.). Then NeutrAvidin was covalently immobilized on the QCM-A plate as follows. To the cleaned bare Au electrode, 500 μL 3,3′-dithiodipropionic acid (4 mM in ethanol solutions) was immobilized the plate for 45 min, after cleaning the 100 μL NHS-EDC solution (both 100 mg mL^–1^ in milli-q water, 1:1) also immobilized the plate for 20 min. After the frequency was stabilization with 500 μL HEPES buff (pH 8.0), 3 μL NeutrAvidin protein solution (10 mg mL^–1^ in milli-q water) was immobilized on the plate for 30 min until the frequency decreased to constant value −2,000. After 2.5 μL of ethanolamine solution was added for 10 min to quench the reaction, the solution was dialyzed to remove unreacted biotin compounds in the HEPES buffer. Next, add 100 μL the biotinylated amylose (10 μg mL^–1^) to anchor on the NeutrAvidin-immobilized QCM-A until the resonance frequency remains constant (±1 Hz) for 30 min (42–46).

### 2.4. Inhibit the enzyme reactions by fucoxanthin on amylose-immobilized QCM-A plates

Direct monitoring is to observe the frequency change after adding different concentrations of the α-amylase solution to QCM-A cells over time. All kinetic processes (enzymatic binding and release, *k*_on_ and *k*_off_, and intramolecular hydrolysis rate, *k*_cat_) of α-amylase hydrolysis of amylose vary with mass. The inhibitory effect of different concentrations (0.005, 0.01, and 0.015 mM in 50% DMSO) of fucoxanthin on α-amylase activity was tested. The bottom stirring device realizes the stirring function, and the stirring speed does not affect the stability and amplitude of the frequency change.

### 2.5. Analysis of enzyme kinetics for α-amylase inhibition by fucoxanthin

The Lineweaver–Burk and Dixon plots were used to determine the kinetic mechanism of α-amylase (47–49). For the Lineweaver–Burk double reciprocal plot, this study determined the enzyme kinetics of α-amylase at various concentrations of amylose (0.2, 0.1, 0.05, and 0.025% in Milli-Q water) as the substrate in the absence or presence of different concentrations of the fucoxanthin (5, 10, 20, 40, and 80 μg mL^–1^ in 50% DMSO). And for the Dixon plot, the inhibition of α-amylase also was obtained in the presence of 0.2, 0.1, 0.05, and 0.025% of amylose substrate. The test concentrations of fucoxanthin in the α-amylase kinetic analysis were as follows: 5, 10, 20, 40, and 80 μg mL^–1^.

### 2.6. Data and statistical analyses

All the assays were performed at least in triplicates, and the data were expressed as mean ± standard deviation (SD). All analyses were performed using the software of SigmaPlot (version 12.5 from Systat Software Inc., San Jose, CA, United States) and GraphPad Prism (version 7.0 from GraphPad Software Inc., San Diego, CA, United States).

## 3. Results and discussion

**FIGURE 2:**
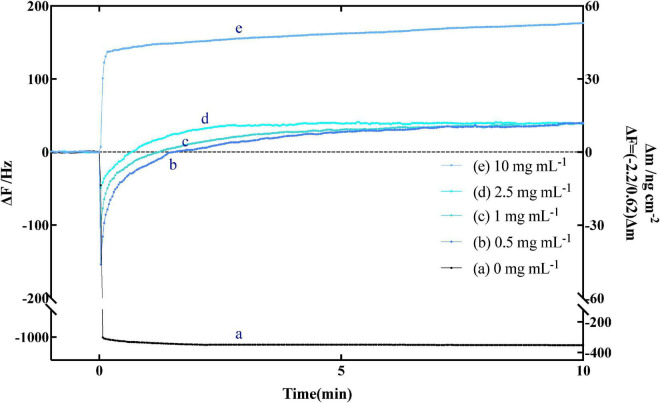
Typical time courses of frequency changes of the amylose–avidin-immobilized quartz crystal microbalance-admittance (QCM-A). (a) α-amylase inactivated with high temperature; (b–e) responding to the addition of different concentrations (0.5, 1, 2.5, and 10 mg mL^–1^) of endo-type α-amylase. All the responding were in pH 7.0 at 37°C.

The curve in Figure 2 shown the typical frequency change of immobilized amylose QCM-A as a function of time, which was carried out in an aqueous solution at pH 7.0. Amylose was fixed on the gold electrode of QCM-A through preliminary experimental preparation. After the frequency was stable, different concentrations of α-amylase, because the enzyme binds to the non-reducing end of amylose as a substrate, the frequency rapidly decreases (the mass increases) in the first few seconds. Subsequently, as the substrate on QCM-A was hydrolyzed by α-amylase, the frequency gradually increased (the mass decreased), and then reached a constant value (Δ*m*) (about −40 ± 5 ng cm^–2^). Since amylose was immobilized on the QCM-A electrode in an amount of 40 ± 5 ng cm^–2^, this clearly indicates that all amylose was immobilized by α-amylase is hydrolyzed and released from the electrode. From the results of curve b, c and d in Figure 2, we can find that even if different concentrations of α-amylase are added, the final constant value will not be greatly affected. It can be determined that this experiment is not related to the added enzyme concentration, but directly related to the amount of amylose immobilized on the electrode.

### 3.1. Transient kinetic analyses on the QCM-A

The curve b–d in Figure 2 shown the hydrolysis of amylose under different concentrations of α-amylase, which can be explained by Equation 1. On the frequency (Δ*F*), the change difference between [ES] complex (Equation 6) and hydrolysate [P] (Equation 7) at different times is more clearly shown. The change of mass m also reflects the above conclusion. And MW_*E*_ and MW_*P*_ in Equation 5 are the molecular weights of α-amylase and hydrolysate, respectively.


(5) $$\Delta \,F=\Delta \,F_{\text{ES}}-\Delta \,F_{\text{p}}=-{\text{MW}}_{\text{E}}\,\left[\text{ES}\right]-\left(-{\text{MW}}_{\text{p}}\,\left[\text{P}\right]\right)$$

(6) $$\Delta \,m=\left[\text{ES}\right]={\left[\text{ES}\right]}_{m\,a\,x}\,\left(1-e^{-t/\tau }\right)-\left[\text{P}\right]\,\left(1-e^{-t/\tau }\right)$$

(7) $$\left[\text{P}\right]=\frac{k_{\text{cat}}}{D_{\text{p}}}\,\int \left[\text{ES}\right]\,\text{d}\,t$$

(8) $$\left[\text{ES}\right]=\text{Z}\,\left(e^{-X\,t}-e^{-Y\,t}\right)$$

(9) $$\left[\text{P}\right]=k_{\text{cat}}\,\text{Z}\,\left(\frac{1-e^{-X\,t}}{X}-\frac{1-e^{-Y\,t}}{Y}\right)$$

**FIGURE 3:**
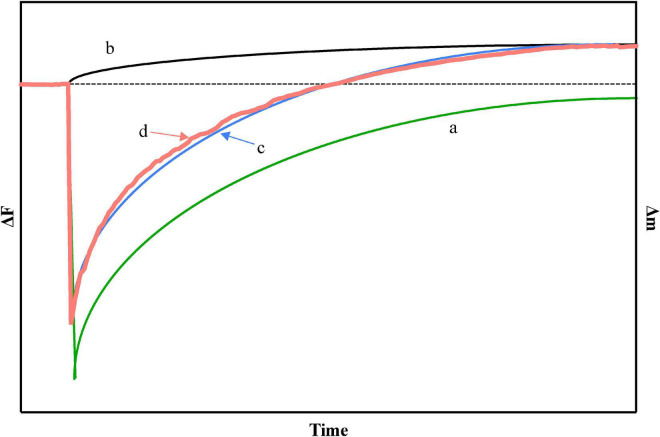
(a) In theory, the relationship between the generation of [ES] and time, (b) in theory, the relationship between the generation of [P] and time, (c) in theory, the relationship with time in the case of simultaneous generation of [ES] and [P], and (d) the actual experimental curve on quartz crystal microbalance-admittance (QCM-A), where [S] = 130 ng cm^–2^.

In order to obtain the theoretical curve, the curve a–c in Figure 3, we use the more extensive Michaelis theory (published by Briggs and Haldane) (50–52) to calculate and fit it by using Kaleidagraph 4.0 (synergy software) in combination with Equations 5, 8, and 9.

Because QCM-A has very good sensitivity, under its experimental conditions, even the initial enzyme amount [E]_0_ with a small concentration is far greater than the ES, P, and *D*_P_ (the degree of polymerization of substrate). Therefore, Equation 2 can be modified to obtain Equation 10:


(10) $$\frac{\text{d}\,\left[\text{ES}\right]}{\text{d}\,t}=k_{\text{on}}\,{\left[\text{E}\right]}_{0}\,\left({\left[\text{S}\right]}_{0}-\left[\text{ES}\right]-\left[\text{P}\right]\right)-\left(k_{\text{off}}+k_{\text{cat}}\right)\,\left[\text{ES}\right]$$

Combined with Equation 2, two-dimensional linear calculus Equation 11 is obtained:


(11) $$\frac{{\text{d}}^{2}\,\left[\text{es}\right]}{\text{d}\,t^{2}}+\left(k_{\text{on}}\,{\left[\text{E}\right]}_{0}+k_{\text{off}}+k_{\text{cat}}\right)\,\frac{\text{d}\,\left[\text{ES}\right]}{\text{d}\,t}+k_{\text{on}}\,k_{\text{cat}}\,{\left[\text{E}\right]}_{0}\,\left[\text{ES}\right]=0$$

When time *t* is 0, ES is not generated and is also 0, and the solution of the above equation is obtained by combining Equations 8, 9.

In order to confirm the parameter values of *X*, *Y*, *Z*, and *k*_cat_, it is necessary to combine the experimental curve with Equations 5, 8, and 9 to obtain the theoretical curve. Then combining Equations 12, 13, *k*_on_ and *k*_off_ can be obtained.

So far, the key values involved in dynamics (*k*_on_, *k*_off_ and *k*_cat_) can be obtained from an experimental curve.


(12) $$\text{X}+\text{Y}\left(=\text{A}\right)k_{\text{on}}{\left[\text{E}\right]}_{0}+k_{\text{off}}+k_{\text{cat}}$$

(13) $$\text{XY}\left(=\text{B}\right)k_{\text{on}}k_{\text{cat}}{\left[\text{E}\right]}_{0}$$

Where,


(14) $$X=\frac{1}{2}\,\left(A-\sqrt{A^{2}-4\,B}\right)$$

(15) $$Y=\frac{1}{2}\,\left(A+\sqrt{A^{2}-4\,B}\right)$$

(16) $$Z=\frac{k_{\text{on}}\,{\left[\text{E}\right]}_{0}\,{\left[\text{S}\right]}_{0}}{Y-X}$$

(17) $$A=k_{\text{on}}\,{\left[\text{E}\right]}_{0}+k_{\text{off}}+k_{\text{cat}}$$

(18) $$B=k_{\text{on}}\,k_{\text{cat}}\,{\left[\text{E}\right]}_{0}$$

We found that the experimental data curve (d) of amylose hydrolysis by α-amylase can be in good agreement with the theoretical curve (c) obtained by equation fitting. By curve fitting method, we obtained the kinetic parameters of *k*_on_, *k*_off_, and *k*_cat_ in Table 1. The values of *K*_d_ and *K*_m_ (*K*_d_ = *k*_off_/*k*_on_ and *K*_m_ = (*k*_off_ + *k*_cat_)/*k*_on_) are also obtained by Equation 4. When the fixed amount of amylose substrate on QCM varied from 20 to 150 ng cm^–2^ and the concentration of α-amylase varied from 0.1 to 5 mg mL^–1^, all kinetic parameters (*k*_on_, *k*_off_, and *k*_cat_) hardly changed within ±12% experimental error.

**TABLE 1:** Kinetic parameters of the endo-type hydrolysis of amylose by α-amylase.

Method	Inhibitor	*k*_on_/M^–1^s^–1^	*k*_off_/s^–1^	*K*_d_/M	*K*_m_/M	*k*_cat_/s^–1^
QCM-A	–	2.6 × 10^3^	5.2 × 10^–4^	0.2 × 10^–6^	2.35 × 10^–3^	6.1
	Fucoxanthin	0.45 × 10^3^	0.27 × 10^–4^	0.6 × 10^–7^	1.78 × 10^–3^	0.8
Michaelis–Menten kinetics	–	–	–	–	1.8 × 10^–3^	8.7
	Fucoxanthin	–	–	–	0.02006 μg mL^–1^	–

### 3.2. Comparison of transient and steady-state kinetics

Using QCM-A to monitor the enzymatic reaction of α-amylase to amylose and perform transient kinetic analysis can effectively obtain the kinetic parameters of each step of the enzymatic reaction, especially the formation of ES complex (*k*_on_), decomposition (*k*_off_), etc. In traditional steady-state kinetics (Michaelis–Menten kinetics) analysis, often only *k*_cat_ and *K*_m_ values (Michaelis constant, (*k*_off_ + *k*_cat_)/*k*_on_) can be obtained. Although different, the relevant kinetic parameters obtained by the QCM method and the values obtained by the steady-state kinetics are relatively consistent (Table 1). It can be seen that under this experimental condition, the immobilization of the substrate hardly affects the hydrolysis reaction.

Calculated according to the equations *K*_m_ = (*k*_off_ + *k*_*cat*_)/*k*_on_ and *K*_d_ = *k*_off_/*k*_on_, it can be known that the *K*_m_ values obtained by QCM-A monitoring and Michaelis-Menten kinetics are 2.35 × 10^–3^ M and 1.8 × 10^–3^ M, respectively, and these *K*_m_ values are much larger than *K*_d_. From the mathematical meaning of the equation, it can be seen that when *k*_off_ ≫ *k*_cat_ occurs, the value of *K*_m_ is composed of the dissociation constant *K*_d_ (Equation 4). In enzyme kinetics, the case of *k*_off_ ≫ *k*_cat_ means that the interaction between the enzyme and the substrate is a state of rapid equilibrium, while the case of *k*_off_ ≪ *k*_cat_ implies that the reaction is almost irreversible. Therefore, the classification of enzyme reaction models should pay special attention to comparing the values of *k*_off_ and *k*_cat_.

The data in Table 1 shown that the *k*_cat_ value (6.1 s^–1^) of this study is much larger than the *k*_off_ value (5.2 × 10^–4^ s^–1^), indicating that the reaction catalyzed by α-amylase to hydrolyze amylose is irreversible, then the *K*_m_ value also does not reflect the dissociation constant (*K*_d_). In this study, the effective kinetic parameters (*k*_off_, *k*_on_, and *k*_cat_) were obtained by the QCM-A method, and then the *K*_d_ value (*k*_off_/*k*_on_) was obtained by calculation. In contrast, the traditional Michaelis–Menten kinetics could not obtain sufficient kinetic parameters, which also cannot reflects the actual dissociation constant. Studies have found that most of the reactions that endonucleases participate in are irreversible reactions. and usually *k*_cat_ is greater than *k*_off_, because endonucleases will continue to hydrolyze specific units of substrates to achieve efficient hydrolysis reactions (53). Therefore, it is essential to monitor the whole process of the response effectively and obtain all the kinetic parameters involved in the reaction for the scientific and accurate analysis of the enzymatic reaction.

### 3.3. Fucoxanthin inhibit α-amylase reactions in the QCM-A plates

**FIGURE 4:**
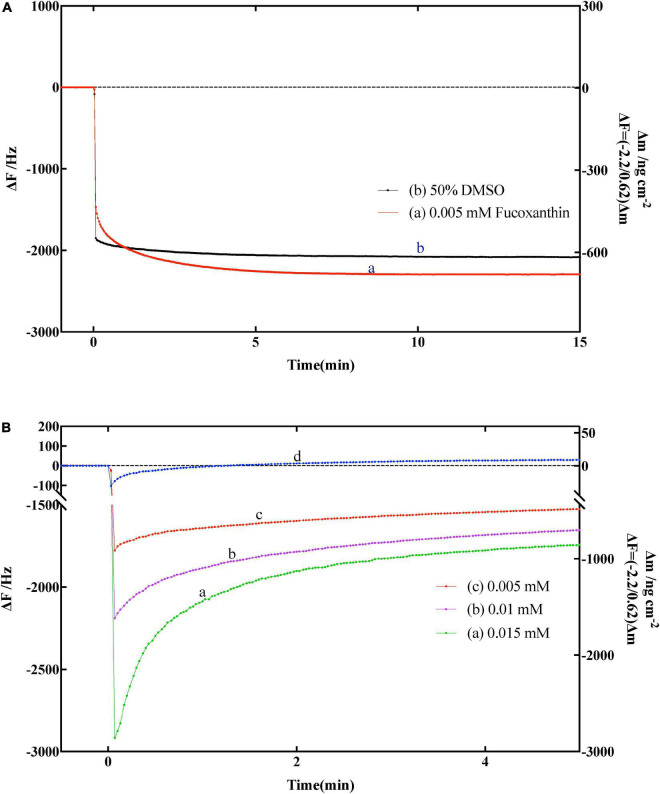
**(A)** Effect of fucoxanthin on frequency changes of the amylose–avidin-immobilized quartz crystal microbalance-admittance (QCM-A). (a) 0.005 nM fucoxanthin in 50% DMSO, (b) 50% DMSO solution. **(B)** Effect of different concentrations fucoxanthin for α-amylase on frequency changes of the amylose–avidin-immobilized QCM-A. (a–c) Different concentrations fucoxanthin in 50% DMSO (0.005, 0.01, 0.015 mM), (d) responding to the addition of 1 mg mL^–1^ α-amylase.

Before determining whether fucoxanthin has an effect on α-amylase, we need to confirm whether fucoxanthin has an effect on the substrate amylose. Therefore, we used the solvent DMSO of fucoxanthin and only fucoxanthin to determine the effect on the substrate amylose on QCM-A. As shown in Figure 4A, the frequency was simply decreased (mass increased) and tended to be stable after a period of time, which indicated that the amylose immobilized on the QCM-A gold electrode did not undergo hydrolysis fracture, bespeaking that fucoxanthin or DMSO had no effect on the substrate structure.

Subsequently, we used fucoxanthin to characterize its effect on the activity of α-amylase. Figure 4B shown the frequency change of QCM-A of immobilized amylose after adding fucoxanthin at different concentrations (0.005, 0.01, and 0.015 mm) in 50% DMSO solution. In the previous data description, since α-amylase hydrolyzes to the non-reducing end of dextran, and the frequency decreases (mass increases) in the first few seconds, and then gradually increases (mass decreases) until reaching a constant value (Figure 4B, d), which corresponds to the hydrolysis of amylose substrate on QCM-A by α-amylase. When different concentrations of fucoxanthin were added to the plate of QCM-A, it was found that the frequency first decreased, and then the frequency gradually increased. However, after the frequency was stable, the constant value was much lower than the constant value using only enzyme. This may be because fucoxanthin has a negative effect on the activity of α-amylase to hydrolyze starch, so that amylose on QCM-A electrode is not catalytically hydrolyzed.

The α-amylase of human salivary was composed of single polypeptide chains with 496 amino acids (54). And the fucoxanthin has two hydroxyl groups and three hydrogen-bond as well as long hydrocarbons conjugated double bonds. Previous researchers proposed that fucoxanthin, as an effective inhibitor of α-amylase or starch blockers, has the characteristics of degrade the activity of α-amylase and decrease the conversion rate of starch (11, 55, 56). This may be stably present in the binding pocket of α-amylase and hydrophobic interactions between fucoxanthin long hydrocarbons with conjugated double bonds and the residues on the α-amylase (57). Meanwhile, the formation of hydrogen bonds between the carboxylate groups (Glu233 and Asp197) in the active site of human salivary α-amylase and OH groups of fucoxanthin, that also may leads to the fucoxanthin against the activity of the α-amylase (54, 56). Furthermore, Hyun et al. (57) found the fucoxanthin in *U. pinnatifida* may have additional hydrogen bonding to might stabilize the enzyme’s open form and potentiate tighter binding to the active site of the enzyme, resulting in more enhance interactions of α-amylase inhibitors.

### 3.4. Inhibitory kinetics of fucoxanthin inhibit α-amylase

It has been reported that the classical Michaelis–Menten equations or Lineweaver–Burk plot has a limitation when fitted the enzymatic hydrolysis curves due to the hydrolysis product changes (47). This drawback occurs when the hydrolysis activity of the enzyme is affected by other inhibitors, resulted in difficulty in predicting the binding and/or cleavage of the substrate, and under this condition, it will be impossible to distinguish which substance (inhibitor or enzyme) on it (39). In contrary, using the QCM method to monitor the hydrolytic enzymatic reaction could accurately analyze the effect of the inhibitor by monitoring the frequency change pattern, the precise kinetic parameters (*k*_on_, *k*_off_, and *K*_d_) obtained during ES complex formation, and *k*_cat_ value during catalytic hydrolysis) (39, 45).

When the enzyme inhibitor is used, there are three experimental conditions of kinetic parameters could be obtained, which explained as follows: (1) while *k*_on_[E]_0_ ≪ *k*_cat_ ≥ *k*_off_, indicates that is an obvious catalytic reaction process. There is a simple increase in frequency, as the mass decreases and shown by the α-amylase (10 mg mL^–1^) curve in Figure 2e; (2) while *k*_on_[E]_0_ ≫ *k*_cat_ ≥ *k*_off_, it showed an obvious enzyme binding process, and the change in QCM-A was a simple decrease in frequency, as an increase in mass (relevant data were not obtained in this study); and (3) while *k*_on_[E]_0_ ≈ *k*_cat_ ≥ *k*_off_, means that the condition is characterized by the competitive enzyme binding and the subsequent catalytic process. The change in QCM-A is started with the frequency first decreases rapidly and then increases, as shown in the curves (b–d) of Figure 2 (45). In the present study, the fucoxanthin reacts with α-amylase like *k*_on_[E]_0_ ≈ *k*_cat_ ≥ *k*_off_, in which the frequency first decreases rapidly, then increases and stabilizes, which may be attributed to the presence of the enzyme. This condition showed that the fucoxanthin has an influence in changing the activity. The curves (a–c) in Figure 4B clearly shown the above reaction. Therefore, various kinetic modes can be analyzed according to multiple situations of frequency change with time. Combining the previous equations, we can know the relationship between the kinetic parameter relaxation time τ^–1^ and [E]_0_ value as follows:


(19) $$\tau^{-1}=k_{\text{on}}\,{\left[\text{E}\right]}_{0}+k_{\text{off}}$$

**FIGURE 5:**
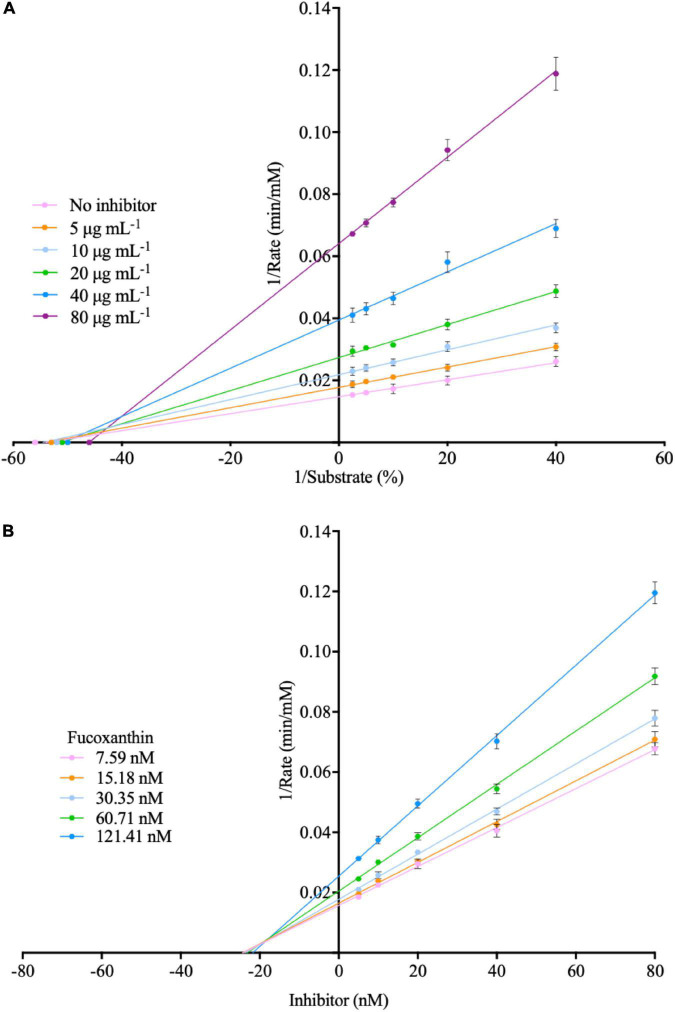
**(A)** Lineweaver-burk plot for the inhibition of fucoxanthin inhibit α-amylase. **(B)** Dixon plot for the inhibition of fucoxanthin inhibit α-amylase.

There is a certain amount of α-amylase in oral saliva. When the human body ingests glucan, it will first undergo a hydrolysis reaction with α-amylase in the saliva. The fucoxanthin used in this study has the property of inhibiting α-amylase, which can effectively reduce the hydrolysis of glucan by α-amylase. Subsequently, we use the Lineweaver–Burk plot and Dixon plot to explain the mode of enzymatic inhibition pattern in Figure 5 (47–49). The kinetic analyses were performed at different concentrations of amylose (0.2, 0.1, 0.05, and 0.025% in milli-q water) and different concentrations of fucoxanthin (5, 10, 20, 40, and 80 μg mL^–1^ in 50% DMSO). In the Lineweaver–Burk plot, the lines of different concentrations of fucoxanthin have similar *y*-intercepts, representing its ability to act as a competitive α-amylase inhibitor, while the lines of fucoxanthin intersect very close to the *y*-axis (the value of the *y*-intercept is close to zero), indicating that the type of inhibition of α-amylase is mixed inhibition and is very similar to non-competitive inhibition. Combined with the above-mentioned QCM-A data and bias of Lineweaver–Burk plot, this study suggests that fucoxanthin has little effect on the substrate amylose, which is negligible compared to the effect on α-amylase activity. Although the binding site of fucoxanthin and α-amylase is not an active site for enzymatic hydrolysis of glucan, it will inhibit or reduce the activity of the enzyme, so that the enzyme cannot hydrolyze glucan. And the *K*_i_ values obtained from the Dixon plotting were 36.25 μM for α-amylase inhibition.

For a long time, elucidating the interaction mechanism between inhibitors and enzymes has been a research hotspot, and various techniques have been used to study the structural characteristics of inhibitors and enzymes, such as 3D molecular docking procedures, cryo-electron microscopy, etc. There are many efficient and fast tools and technologies, but there are also some shortcomings. For example, molecular docking technology, which is a prediction method, sometimes cannot fully reflect the real reaction changes (58, 59); and cryo-electron microscopy technology is efficient and fast, but the equipment is too large. Expensive, sample preparation is too complicated, etc. The QCM technology used in this study can obtain precise kinetic parameters, thereby providing real and accurate data for kinetic studies.

## 4. Conclusion

In this study, we employed the QCM-A instrument to explain each step of the reaction between α-amylase and amylose and the inhibition effect of seaweed fucoxanthin against α-amylase in amylose hydrolysis, which is very important to fully understand its kinetic reaction mechanism. Further understanding of fucoxanthin- α-amylase interactions plays an important role in promoting its commercial development. Compared to the conventional method, the Michaelis–Menten equation, the QCM-A technique can observe and quantify every step of the catalytic hydrolysis reaction, especially the formation and decay of ES complexes. Our experimental work shown that the addition of various concentrations of fucoxanthin (0.005, 0.01, and 0.015 mM) could give a negative effect on the activity of α-amylase to hydrolyze starch, so that amylose on QCM-A electrode is not catalytically hydrolyzed. These observations prove that the QCM-A is suitable to investigate the inhibitory effect of fucoxanthin against α-amylase. In addition, this experimental technique will be applicable for any other type of polysaccharide degradation studies, any active enzymes involving hydrolysis or carbohydrates, can be tested, and characterized using this experimental technique. Furthermore, the QCM-A technology is also useful for the development of novel inhibitors of digestive enzymes.

## Data availability statement

The original contributions presented in this study are included in the article/supplementary material, further inquiries can be directed to the corresponding authors.

## Author contributions

SY: conceptualization, methodology, validation, investigation, formal analysis, data curation, and writing—original draft and editing. ES: writing—review and editing. LN: methodology and writing—review and editing. MS and YL: methodology. TH: funding acquisition, methodology, project administration, and supervision. All authors contributed to the article and approved the submitted version.

## References

[1] Liu J Ren Z Qiang H Wu J Shen M Zhang L Trends in the incidence of diabetes mellitus: results from the Global Burden of Disease Study 2017 and implications for diabetes mellitus prevention. *BMC Public Health.* (2020) 20:1415. 10.1186/s12889-020-09502-x 32943028 PMC7500018

[2] Harding J Pavkov M Magliano D Shaw J Gregg E. Global trends in diabetes complications: a review of current evidence. *Diabetologia.* (2019) 62:3–16. 10.1007/s00125-018-4711-2 30171279

[3] Kalita D Holm D LaBarbera D Petrash J Jayanty S. Inhibition of alpha-glucosidase, alpha-amylase, and aldose reductase by potato polyphenolic compounds. *PLoS One.* (2018) 13:e0191025. 10.1371/journal.pone.0191025 29370193 PMC5784920

[4] Teng H Chen L. Alpha-glucosidase and alpha-amylase inhibitors from seed oil: a review of liposoluble substance to treat diabetes. *Crit Rev Food Sci Nutr.* (2017) 57:3438–48. 10.1080/10408398.2015.1129309 26854322

[5] Lopes G Andrade P Valentao P. Phlorotannins: towards new pharmacological interventions for diabetes mellitus type 2. *Molecules.* (2016) 22:56. 10.3390/molecules22010056 28042834 PMC6155720

[6] Dirir A Daou M Yousef A Yousef L. A review of alpha-glucosidase inhibitors from plants as potential candidates for the treatment of type-2 diabetes. *Phytochem Rev.* (2022) 21:1049–79. 10.1007/s11101-021-09773-1 34421444 PMC8364835

[7] Haq F Siraj A Ameer M Hamid T Rahman M Khan S Comparative review of drugs used in diabetes mellitus—new and old. *J Diabetes Mellitus.* (2021) 11:115–31. 10.4236/jdm.2021.114009

[8] Hedrington M Davis S. Considerations when using alpha-glucosidase inhibitors in the treatment of type 2 diabetes. *Expert Opin Pharmacother.* (2019) 20:2229–35. 10.1080/14656566.2019.1672660 31593486

[9] Khaw Y Yusoff F Tan H Noor Mazli N Nazarudin M Shaharuddin N The critical studies of fucoxanthin research trends from 1928 to June 2021: a bibliometric review. *Mar Drugs.* (2021) 19:606. 10.3390/md19110606 34822476 PMC8623609

[10] Mandotra S Upadhyay A Ahluwalia A. *Algae: Multifarious Applications for a Sustainable World.* Berlin: Springer Nature (2020). 10.1007/978-981-15-7518-1

[11] Yin S Shibata M Tomoaki H. Extraction of bioactive compounds from stems of *Undaria pinnatifida*. *Food Sci Technol Res.* (2019) 25:765–73. 10.3136/fstr.25.765

[12] Sivagnanam S Yin S Choi J Park Y Woo H Chun B. Biological properties of fucoxanthin in oil recovered from two brown seaweeds using supercritical Co2 extraction. *Mar Drugs.* (2015) 13:3422–42. 10.3390/md13063422 26035021 PMC4483637

[13] Ojulari O Lee S Nam J. Therapeutic effect of seaweed derived xanthophyl carotenoid on obesity management; overview of the last decade. *Int J Mol Sci.* (2020) 21:2502. 10.3390/ijms21072502 32260306 PMC7177665

[14] Zarekarizi A Hoffmann L Burritt D. Approaches for the sustainable production of fucoxanthin, a xanthophyll with potential health benefits. *J Appl Phycol.* (2018) 31:281–99. 10.1007/s10811-018-1558-3

[15] Xie X Chen C Fu X. Screening α-glucosidase inhibitors from four edible brown seaweed extracts by ultra-filtration and molecular docking. *LWT Food Sci Technol.* (2021) 138:110654. 10.1016/j.lwt.2020.110654

[16] Zaharudin N Salmean A Dragsted L. Inhibitory effects of edible seaweeds, polyphenolics and alginates on the activities of porcine pancreatic alpha-amylase. *Food Chem.* (2018) 245:1196–203. 10.1016/j.foodchem.2017.11.027 29287342

[17] Maeda H Hosokawa M Sashima T Murakami-Funayama K Miyashita K. Anti-obesity and anti-diabetic effects of fucoxanthin on diet-induced obesity conditions in a murine model. *Mol Med Rep.* (2009) 2:897–902. 10.3892/mmr_00000189 21475918

[18] Yin S Niu L Shibata M Liu Y Tomoaki H. Optimization of fucoxanthin extraction obtained from natural by-products from *Undaria pinnatifida* stem using supercritical Co2 extraction method. *Front Nutr.* (2022) 9:981176. 10.3389/fnut.2022.981176 36245524 PMC9558218

[19] Oliyaei N Moosavi-Nasab M Tamaddon A Tanideh N. Antidiabetic effect of fucoxanthin extracted from *Sargassum angustifolium* on streptozotocin-nicotinamide-induced type 2 diabetic mice. *Food Sci Nutr.* (2021) 9:3521–9. 10.1002/fsn3.2301 34262712 PMC8269564

[20] Maeda H Hosokawa M Sashima T Miyashita K. Dietary combination of fucoxanthin and fish oil attenuates the weight gain of white adipose tissue and decreases blood glucose in obese/diabetic Kk-Ay mice. *J Agric Food Chem.* (2007) 55:7701–6. 10.1021/jf071569n 17715888

[21] Nishikawa S Hosokawa M Miyashita K. Fucoxanthin promotes translocation and induction of glucose transporter 4 in skeletal muscles of diabetic/obese Kk-Ay mice. *Phytomedicine.* (2012) 19:389–94. 10.1016/j.phymed.2011.11.001 22305278

[22] Hosokawa M Miyashita T Nishikawa S Emi S Tsukui T Beppu F Fucoxanthin regulates adipocytokine mrna expression in white adipose tissue of diabetic/obese Kk-Ay mice. *Arch Biochem Biophys.* (2010) 504:17–25. 10.1016/j.abb.2010.05.031 20515643

[23] Procházka V Kulha P Izsák T Ukraintsev E Varga M Jirásek V Detection of globular and fibrillar proteins by quartz crystal microbalance sensor coated with a functionalized diamond thin film. *Appl Surf Sci.* (2022) 589:153017. 10.1016/j.apsusc.2022.153017

[24] Jandas P Prabakaran K Luo J Holaday MG. Effective utilization of quartz crystal microbalance as a tool for biosensing applications. *Sens Actuators A Phys.* (2021) 331:113020. 10.1016/j.sna.2021.113020

[25] Mosley R Talarico M Byrne M. Recent applications of QCM-D for the design, synthesis, and characterization of bioactive materials. *J Bioact Compat Polym.* (2021) 36:261–75. 10.1177/08839115211014216

[26] Migon D Wasilewski T Suchy D. Application of QCM in peptide and protein-based drug product development. *Molecules.* (2020) 25:3950. 10.3390/molecules25173950 32872496 PMC7504752

[27] Johannsmann D. The quartz crystal microbalance in soft matter research. *Soft and Biological Matter.* (Switzerland: Springer Cham) (2015). 10.1007/978-3-319-07836-6

[28] Sunar M editor. Piezoelectric materials. *Comprehensive Energy Systems.* (Amsterdam: Elsevier) (2018). 10.1016/B978-0-12-809597-3.00248-0

[29] Furusawa H Sekine T Ozeki T. Hydration and viscoelastic properties of high- and low-density polymer brushes using a quartz-crystal microbalance based on admittance analysis (QCM-A). *Macromolecules.* (2016) 49:3463–70. 10.1021/acs.macromol.6b00035

[30] Tomoaki H Nattawut P Shibata M Sakiyama T. Monitoring of adsorption behaviors of bovine serum albumin onto a stainless steel surface by the quartz crystal microbalance based on admittance analysis. *Biosci Biotech Biochem.* (2017) 81:783–9. 10.1080/09168451.2017.1281724 28110631

[31] Cornelio V Pedroso M Afonso A Fernandes J da Silva M Faria R New approach for natural products screening by real-time monitoring of hemoglobin hydrolysis using quartz crystal microbalance. *Anal Chim Acta.* (2015) 862:86–93. 10.1016/j.aca.2015.01.003 25682432

[32] Furusawa H Tsuyuki Y Takahashi S Okahata Y. In situ monitoring of structural changes during formation of 30s translation initiation complex by energy dissipation measurement using 27-MHz quartz-crystal microbalance. *Anal Chem.* (2014) 86:5406–15. 10.1021/ac500487b 24794712

[33] Li X Song S Shuai Q Pei Y Aastrup T Pei Y Real-time and label-free analysis of binding thermodynamics of carbohydrate-protein interactions on unfixed cancer cell surfaces using a QCM biosensor. *Sci Rep.* (2015) 5:14066. 10.1038/srep14066 26369583 PMC4570189

[34] Mori T Shibata M Nihira T Mikami B Okahata Y. Kinetic monitoring of site-directed mutational β-amylase catalysis on a 27-MHz QCM. *J Mol Catal B Enzym.* (2012) 82:121–6. 10.1016/j.molcatb.2012.05.019

[35] Gray C Weissenborn M Eyers C Flitsch S. Enzymatic reactions on immobilised substrates. *Chem Soc Rev.* (2013) 42:6378–405. 10.1039/c3cs60018a 23579870

[36] Furusawa H Takano H Okahata Y. Transient kinetic studies of Ph-dependent hydrolyses by exo-type carboxypeptidase P on a 27-MHz quartz crystal microbalance. *Anal Chem.* (2008) 80:1005–11. 10.1021/ac702290z 18211097

[37] Chen Q Xu S Liu Q Masliyah J Xu Z. QCM-D study of nanoparticle interactions. *Adv Colloid Interface Sci.* (2016) 233:94–114. 10.1016/j.cis.2015.10.004 26546115

[38] Nishino H Murakawa A Mori T Okahata Y. Kinetic studies of amp-dependent phosphorolysis of amylopectin catalyzed by phosphorylase B on a 27 MHz quartz-crystal microbalance. *J Am Chem Soc.* (2004) 126:14752–7. 10.1021/ja046583k 15535699

[39] Nihira T Mizuno M Tonozuka T Sakano Y Mori T Okahata Y. Kinetic studies of site-directed mutational isomalto-dextranase-catalyzed hydrolytic reactions on a 27 MHz quartz-crystal microbalance. *Biochemistry.* (2005) 44:9456–61. 10.1021/bi050079q 15996100

[40] Jafari-Aghdam J Khajeh K Ranjbar B Nemat-Gorgani M. Deglycosylation of glucoamylase from *Aspergillus niger*: effects on structure, activity and stability. *Biochim Biophys Acta.* (2005) 1750:61–8. 10.1016/j.bbapap.2005.03.011 15886078

[41] Sauerbrey G. Verwendung von schwingquarzen zur wägung dünner schichten und zur mikrowägung. *Z Angew Phys.* (1959) 155:206–22. 10.1007/BF01337937

[42] Ozeki T Morita M Yoshimine H Furusawa H Okahata Y. Hydration and energy dissipation measurements of biomolecules on a piezoelectric quartz oscillator by admittance analyses. *Anal Chem.* (2007) 79:79–88. 10.1021/ac060873x 17194124

[43] Sun X Faucher K Houston M Grande D Chaikof E. Design and synthesis of biotin chain-terminated glycopolymers for surface glycoengineering. *J Am Chem Soc.* (2002) 124:7258–9. 10.1021/ja025788v 12071720

[44] Shinohara Y Sota H Gotoh M Hasebe M Tosu M Nakao J Bifunctional labeling reagent for oligosaccharides to incorporate both chromophore and biotin groups. *Anal Chem.* (1996) 68:2573–9. 10.1021/ac960004f 8694260

[45] Nishino H Nihira T Mori T Okahata Y. Direct monitoring of enzymatic glucan hydrolysis on a 27-MHz quartz-crystal microbalance. *J Am Chem Soc.* (2004) 126:2264–5. 10.1021/ja0361805 14982404

[46] Okahata Y. *A Quartz Crystal Microbalance Method for Biosensing: To Applications from the Principles (Ks Specialty Chemicals Manual).* Tokyo: Kodansha (2013).

[47] Lineweaver H Burk D. The determination of enzyme dissociation constants. *J Am Chem Soc.* (2002) 56:658–66. 10.1021/ja01318a036

[48] Cornish-Bowden A. A simple graphical method for determining the inhibition constants of mixed, uncompetitive and non-competitive inhibitors. *Biochem J.* (1974) 137:143. 10.1042/bj1370143 4206907 PMC1166095

[49] Dixon M. The determination of enzyme inhibitor constants. *Biochem J.* (1953) 55:170. 10.1042/bj0550170 13093635 PMC1269152

[50] Noor E Flamholz A Liebermeister W Bar-Even A Milo RA. Note on the kinetics of enzyme action: a decomposition that highlights thermodynamic effects. *FEBS Lett.* (2013) 587:2772–7. 10.1016/j.febslet.2013.07.028 23892083

[51] Chance B. The kinetics of the enzyme-substrate compound of peroxidase. *J Biol Chem.* (1943) 151:553–77. 10.1016/S0021-9258(18)44929-0 10218104

[52] Briggs G Haldane J. A note on the kinetics of enzyme action. *Biochem J.* (1925) 19:338–9. 10.1042/bj0190338 16743508 PMC1259181

[53] Wang Y Kansou K Pritchard J Zwart A Saulnier L Ral J. Beyond amylose content, selecting starch traits impacting in vitro alpha-amylase degradability in a wheat magic population. *Carbohydr Polym.* (2022) 291:119652. 10.1016/j.carbpol.2022.119652 35698355

[54] Gumucio D Wiebauer K Caldwell R Samuelson L Meisler M. Concerted evolution of human amylase genes. *Mol Cell Biol.* (1988) 8:1197–205. 10.1128/mcb.8.3.1197-1205.1988 2452973 PMC363264

[55] Din N Mohd Alayudin A Sofian-Seng N Rahman H Mohd Razali N Lim S Brown algae as functional food source of fucoxanthin: a review. *Foods.* (2022) 11:2235. 10.3390/foods11152235 35954003 PMC9368577

[56] Kawee-Ai A Kim A Kim S. Inhibitory activities of microalgal fucoxanthin against α-amylase, α-glucosidase, and glucose oxidase in 3T3-L1 cells linked to type 2 diabetes. *J Oceanol Limnol.* (2019) 37:928–37. 10.1007/s00343-019-8098-9

[57] Jung H Islam M Lee C Jeong H Chung H Woo H Promising antidiabetic potential of fucoxanthin isolated from the edible brown algae *Eisenia bicyclis* and *Undaria pinnatifida*. *Fish Sci.* (2012) 78:1321–9. 10.1007/s12562-012-0552-y

[58] Chen G Seukep A Guo M. Recent advances in molecular docking for the research and discovery of potential marine drugs. *Mar Drugs.* (2020) 18:545. 10.3390/md18110545 33143025 PMC7692358

[59] Pinzi L Rastelli G. Molecular docking: shifting paradigms in drug discovery. *Int J Mol Sci.* (2019) 20:4331. 10.3390/ijms20184331 31487867 PMC6769923

